# Advice Wanted

**Published:** 1861-02

**Authors:** 


Advice Wanted.—Requests for medical advice are occasionally received by this journal from persons who are evidently not members of the profession, but who assume to be, perhaps for the purpose of getting it gratuitously. Such requests remain, of course, unanswered. As an illustration of this practice the subjoined, which has just been received, is presented:

"Mr. Editors,

A patient of mine is sufrine from the followinge aperancies he will take a ake in the stomake like a stomake ake until he throes of his vittals and then he gets better, and can eat until the stomake ake takes him againe and serves him the same way againe until nothing wont stay down—I relieved him onst best by mustird and number 6 also head ake bake ake in the loins and fever until it comes up—Will you anser to corespondance the best proscriptoine."—*Medical and Surgical Reporter.*

